# Comparative analysis of complete chloroplast genomes of *Cousinia* (Asteraceae) species

**DOI:** 10.3389/fpls.2025.1522950

**Published:** 2025-04-29

**Authors:** Boburbek Karimov, Sh. Komiljon Tojibaev, Dilnoza Azimova, Ziyoviddin Yusupov, Lufeng Liu

**Affiliations:** ^1^ Institute of Botany, Academy of Sciences of Uzbekistan, Tashkent, Uzbekistan; ^2^ Department of Biology Teaching Methodology, Jizzakh State Pedagogical University, Jizzakh, Uzbekistan; ^3^ College of Resources and Environment, Yunnan Agricultural University, Kunming, China

**Keywords:** *Cardueae tribe*, plastid genome, comparative analysis, phylogeny, anther appendages

## Abstract

The study focused on analyzing the chloroplast genome structure and investigating the phylogenetic relationships among six species of the *Cousinia* genus. Within the Asteraceae family, the complete chloroplast genome sequences of six *Cousinia* species, ranging from 152,553 to 152,619 bp. The chloroplast genomes exhibit a characteristic quadripartite structure. The gene order is largely conserved across the genus, with an exception in the small single copy region, where a reverse orientation is observed in comparison to *Cousinia thomsonii*. A total of 131 genes were annotated, including 87 protein-coding genes, 36 tRNA genes, and 8 rRNA genes, with 18 genes showing duplication. Notably, 16 genes contain introns, with *ycf3* and *clpP* carrying two introns each. Nucleotide diversity analysis revealed 412 polymorphic sites across 152,892 nucleotides in six *Cousinia* species. Higher nucleotide polymorphism levels were found in *trnE-UUC – rpoB, trnL-UAA – trnF-GAA – ndhJ, rbcL*, and *ycf1* genomic regions, indicating possible genomic loci for species differentiation. Phylogenetic analysis using complete chloroplast genomes, demonstrated the genus *Cousinia*’s phylogenetic placement within the Cardueae tribe, forming distinct clades that align with its traditional sectional classification. The Arctiinae subtribe, containing *Cousinia*, forms a monophyletic group with *Arctium lappa*, while Saussureinae were found to be polyphyletic. The findings suggest that while morphological traits are valuable in taxonomy, they may provide limited resolution compared to the more comprehensive phylogenetic insights obtained from chloroplast genome analysis.

## Introduction

*Cousinia* Cass. (Asteraceae, Cardueae) is a large genus, being the 50th largest genera among flowering plants. It is represented by approximately 671 species (Frodin, 2004; Ulukuş and Tugay, 2020; Uysal et al., 2022; POWO, 2025). The species of *Cousinia* are typically found in arid regions, with a preference for upland vegetation. They are mainly distributed in the mountainous areas of Turkey, Iran, Afghanistan and Central Asia (Knapp, 1987). Central Asia, home to ca. 260 species, is considered the most significant region for these plants (Tscherneva, 1993).

*Cousinia* was first described by Cassini (1827). de Candolle, (1824) listed 34 species of *Cousinia*, organized into three categories according to the morphology of their capitula. In an early effort to comprehensively classify the genus *Cousinia*, von Bunge (1865) categorized 126 species of *Cousinia* into 23 sections, primarily based on growth habit and capitular features, such as the texture of receptacular bristles, shape of receptacular bracts, corolla color, color of the anther tube, and whether or not hairs were present on the anther tube. Bunge’s classification served as a foundation for later studies, including Boissier’s Flora Orientalis (1875) and its supplement (1888), which documented 141 species of *Cousinia* organized into 14 sections. The next significant classification was by Winkler in 1892 (Winker, 1892), who emphasized capitulum morphology for taxonomy like Bunge’s classification. In a notable divergence, Kuntze (1891) proposed merging *Cousinia* with the genus *Arctium* L. Winkler’s work (1892, 1897) expanded to include 267 species divided into three subgenera (*Oligochaete, Dichacantha*, and *Eucousinia*). Bunge’s approach laid the groundwork for a more natural classification system as later elaborated by Rechinger (1953, 1972) and Tscherneva (1962, 1988). Rechinger’s Flora Iranica (1972) included over 350 species across 58 sections, addressing the flora of the Iranian Plateau, Turkmenistan, Afghanistan, and the mountainous regions of Pakistan. Similarly, Tscherneva’s treatment (1962, 1988) for the Flora of the USSR cataloged around 260 species in 50 sections, covering regions of Central Asia and the Caucasus. According to Sennikov (2010), the sections *Alpinae* Bunge (*Carduncellus* (Juz.) Rech. f.) and *Subappendiculatae* Tschern. were discovered to be varied in their involucre structure, anther appendage shape, and basal leaves. Consequently, the classification now includes three sections instead of two, with a new section, *Tianschanicae* Sennikov, being distinguished from *Alpinae*. Additionally, *Cousinia knorringiae* has been reassigned from the *Alpinae* section to the *Subappendiculatae* section, while *Cousinia omissa* and *Cousinia subappendiculata* have been moved from *Subappendiculatae* to *Tianschanicae.*

Molecular studies started at the end of the 1990s. The phylogeny of the Cardueae tribe, studied by Häffner and Hellwig (1999) using ITS sequence data and later supported by Garcia-Jacas et al. (2002) through ITS and *matK* data, demonstrated a close relationship among the genera *Cousinia*, *Arctium*, *Saussurea*, and *Jurinea*. The molecular analysis of the *Arctium-Cousinia* complex by Susanna et al. (2003) identified two main lineages: the Arctioid clade, which includes *Arctium* s.str. and certain *Cousinia* subgenera (*Hypacanthodes, Cynaroides, Hypacanthium, and Schmalhausenia*), and the Cousinioid clade, consisting of *Cousinia* subg. *Cousinia*. This classification was supported by subsequent studies (Susanna et al., 2006; López-Vinyallonga et al., 2009). In 2011, López-Vinyallonga et al. transferred all Arctioid species to *Arctium* based on analyses of ITS, *trnL-trnF*, and *matK* sequences. Mehregan and Kadereit (2009) investigated potential hybridization among 214 species of *Cousinia* and related genera, concluding that there was no evidence of hybridization between *Cousinia* s.str. and other clades within the *Arctium-Cousinia* complex, nor between annual and perennial species of *Cousinia* s.str. Atazadeh et al. (2021) focused on species delineation within the sections *Cynaroideae* and *Platyacanthae*, but their ITS-based phylogenetic tree did not separate these sections. Kalouti et al. (2022) examined the monophyly of *Cousinia* sect. *Stenocephalae*, along with sect. *Albidae* and *Cousinia*, finding that none of these sections are monophyletic based on ITS data.

While various studies have used genetic sequence data from nuclear ITS regions and short chloroplast markers to explore relationships within the genus (Häffner and Hellwig, 1999; Garcia-Jacas et al., 2002; Susanna et al., 2003, 2006; López-Vinyallonga et al., 2009, 2011; Mehregan and Kadereit, 2009; Atazadeh et al., 2021; Kalouti et al., 2022), none have focused on the complete structure of the chloroplast (cp) genome, and only one cp genome of *Cousinia* is currently available in GenBank (Accession No. PP525141).

To address these gaps in understanding the evolutionary relationships within *Cousinia*, we sequenced the cp genomes of six *Cousinia* species. These include *Cousinia rhodantha* Kult. (Southwestern Pamir-Alay), *C. pseudodshizakensis* Tschern. & Vved. (Northwestern Pamir-Alay), *C. proxima* Juz. (Gissar Range), *C. subcandicans* Tschern. (Gissar Range), *C. orthacantha* Tschern. (Southwestern Pamir-Alay, Afghanistan), and *C. rotundifolia* C.Winkl. (Pamir-Alay), exhibit highly restricted distributions (Tscherneva, 1962), emphasizing their endemism and conservation importance. These species play crucial ecological roles in their native mountainous habitats, contributing to soil stabilization, supporting pollinators, and maintaining local biodiversity.

The primary objectives of our study were: 1) to analyze the cp genome structures of *Cousinia* species, 2) to identify the most divergent regions that can aid in species differentiation, 3) to make phylogenetic inferences to determine the position of *Cousinia*.

## Materials and methods

### Plant materials

**Figure 1:**
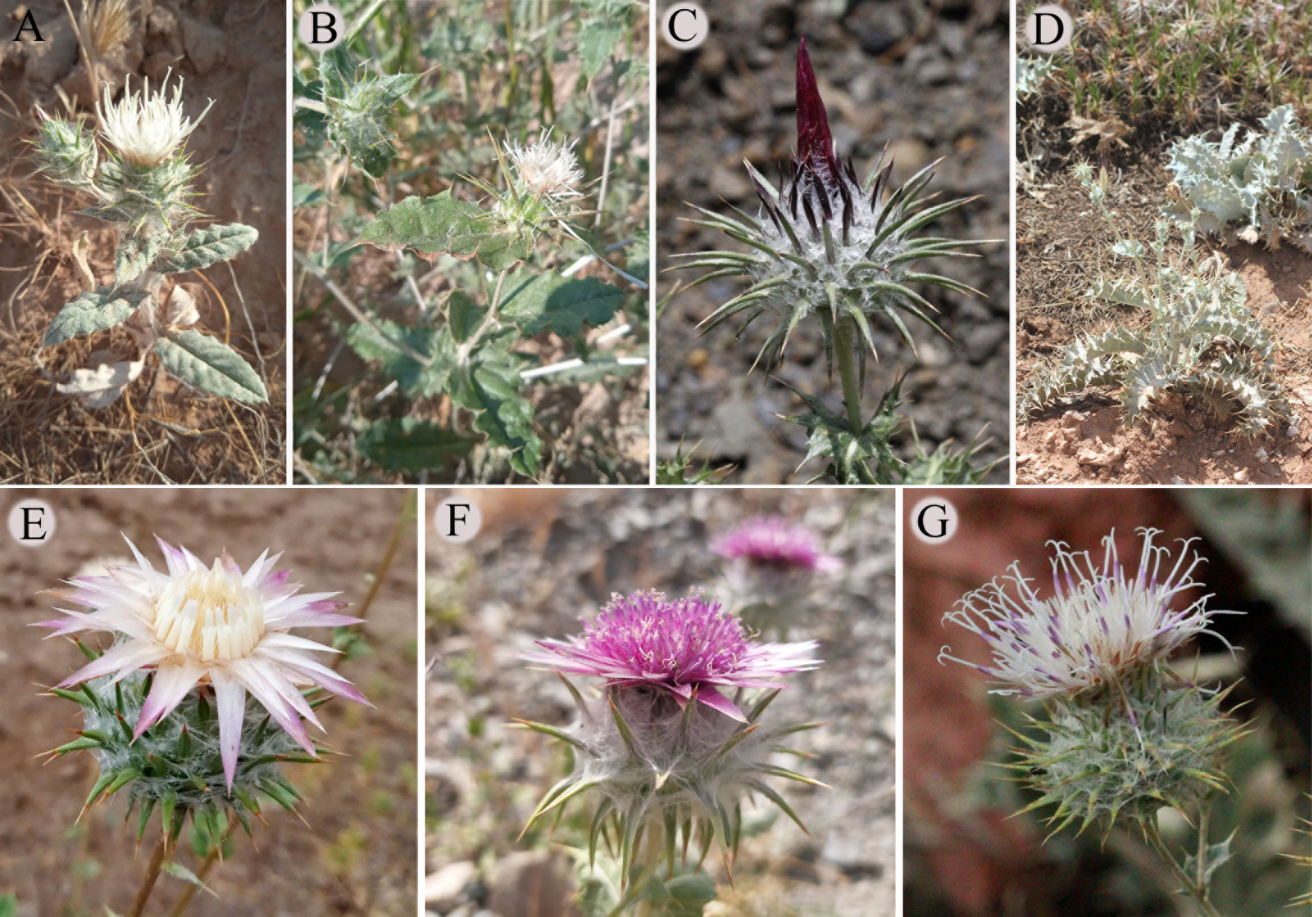
Collected species of *Cousinia* in this study. **(A)** *Cousinia orthacantha* (desert) **(B)** *Cousinia orthacantha* (agricultural area) **(C)** *Cousinia rhodantha*, **(D)** *Cousinia subcandicans*, **(E)** *Cousinia rotundifolia*, **(F)** *Cousinia pseudodshizakensis*, **(G)** *Cousinia proxima*.

The study species represented three distinct sections within the genus *Cousinia*: sect. *Alpinae* (syn. *Cousinia* section *Carduncellus*) (*Cousinia pseudodshizakensis*, *C. rhodantha* and *C. rotundifolia*), sect. *Homalochaete* (*C. subcandicans* and *C. proxima*) and sect. *Dichotomae* (*C. orthacantha* collected in two populations). (**Figure 1**). All the species were collected from Uzbekistan. The herbarium specimens have been deposited at the National Herbarium of Uzbekistan (TASH) (**Supplementary Table S1**).

### Sequencing, assembly, and annotation

Genomic DNA was isolated from leaf samples using the DP305 Plant Genomic DNA kits from Tiangen, Beijing, China, following the provided protocol. For library preparation, the NEBNext^®^ UltraTM DNA Library Prep Kit for Illumina from NEB, USA (Catalog: E7370L) was utilized, following the manufacturer’s recommendations. Index codes were incorporated into each sample. The genomic DNA underwent sonication to fragment it into pieces of approximately 350 base pairs. The resulting DNA fragments were subjected to end polishing, A-tailing, and ligated with full-length adapters suitable for Illumina sequencing. Subsequent PCR amplification was performed. The PCR products were purified using the AMPure XP system from Beverly, USA. The quality of the libraries was evaluated using the Agilent 5400 system from Agilent, USA, and their concentration was determined by QPCR (1.5 nM). The qualified libraries were combined and sequenced on Illumina platforms using the PE150 strategy at Novogene Bioinformatics Technology Co., Ltd in Beijing, China, according to the required library concentrations and data volume. In an investigation of six *Cousinia* species, paired-end sequencing produced between 7,890,409 reads (*C. pseudodshizakensis*) and 20,105,500 reads (*C. subcandicans*), with each read comprising 150 base pairs. Based on an estimated genome size of 150 kilobases, this corresponds to a sequencing depth ranging from approximately 7,890× to 20,105×. *De novo* assembly of the cp genomes for the six *Cousinia* species was performed using NOVOPlasty v4.3.4 (Dierckxsens et al., 2017). Clean paired-end reads in FASTQ format were used as input. During the time of our analysis, the cp genome sequence of *Cousinia thomsonii* (PP525141) was not available in the NCBI database. Consequently, *Arctium lappa* (NC042724) was chosen as a reference for assembly and annotation due to its availability and taxonomic placement within the Arctiinae subtribe. The assemblies produced circular cp genomes.

Gene annotation was carried out using Geneious v.9.0.2, with *Arctium lappa* set as the reference. Manual inspection was performed to verify the start and stop codons and the boundaries of introns and exons for protein-coding genes (Kearse et al., 2012).

The SRA (Sequence Read Archive) data associated with the PRJNA1124612 BioProject has been deposited in the GenBank database (**Supplementary Table S2**).

### Genome structure analyses

The identification of cp simple sequence repeats (SSRs) was performed using the MIcroSAtellite (https://webblast.ipk-gatersleben.de/misa/) web tool (Beier et al., 2017). The search parameters were set to detect perfect mono-, di-, tri-, tetra-, penta-, and hexa nucleotide motifs with a minimum repeat count of 10, 5, 4, 3, 3, and 3, respectively. Additionally, the REPuter web tool (https://bibiserv.cebitec.uni-bielefeld.de/reputer) (Kurtz et al., 2001) was employed to identify various types of repeats, including forward, reverse, palindrome, and complement sequences, within the cp genomes. The repeat identification settings used were: a hamming distance of three, a minimum repeat size of 30 base pairs, and a maximum computed repeat size of 90 base pairs.

### Genome comparison and nucleotide variation analysis

The OGDRAWv1.1 online tool (https://chlorobox.mpimp-golm.mpg.de/OGDraw.html) (Lohse et al., 2007) was employed for the graphical representation of the cp genome. The nucleotide variability (Pi) values were obtained with the DnaSP v.6.12.03 software (Rozas et al., 2017). A window length of 1500 base pairs and a step size of 700 base pairs were used for this analysis. The IR region boundaries, as well as the genomic regions, were visualized and analyzed using the IRscope online analysis tool (https://irscope.shinyapps.io/irapp/) (Amiryousefi et al., 2018).

### Phylogenetic analyses

The newly sequenced cp genomes and nuclear ribosomal DNA (nrDNA) ITS sequences of six *Cousinia* species, along with 39 plastome sequences and 76 nrDNA ITS sequences from the Cardueae tribe obtained from NCBI (**Supplementary Table S3**), were utilized to construct a phylogenetic tree. *Famatinanthus decussatus* (Hieron.) Ariza & S.E. Freire was designated as the outgroup (Herrando-Moraira et al., 2019). The MAFFT software (Katoh and Standley, 2013) was used for the alignment of complete cp genomes. Bayesian analyses were performed using MrBayes v.3.2 (Ronquist et al., 2011). The best-fitting models of nucleotide substitutions, selected based on the Akaike information criterion (AIC) in jModelTest v.2.1.4 (Darriba et al., 2012), were employed in this analysis. Maximum likelihood (ML) analyses were carried out using RAxML v.8.0 (Stamatakis, 2014) with the best-fit GTR + G model and 1000 bootstrap replicates.

### Microscopic examination of anther appendages

We investigated the morphology of anther appendages in 20 species from three sections (*Alpinae*, *Homalochaete*, *Dichotomae*) of the *Cousinia* genus using microscopy. Specimens were sourced from the National Herbarium of Uzbekistan and the Herbarium of the Institute of Botany, Plant Physiology, and Genetics at the National Academy of Sciences of Tajikistan.

## Results

### Chloroplast genome features

**Figure 2:**
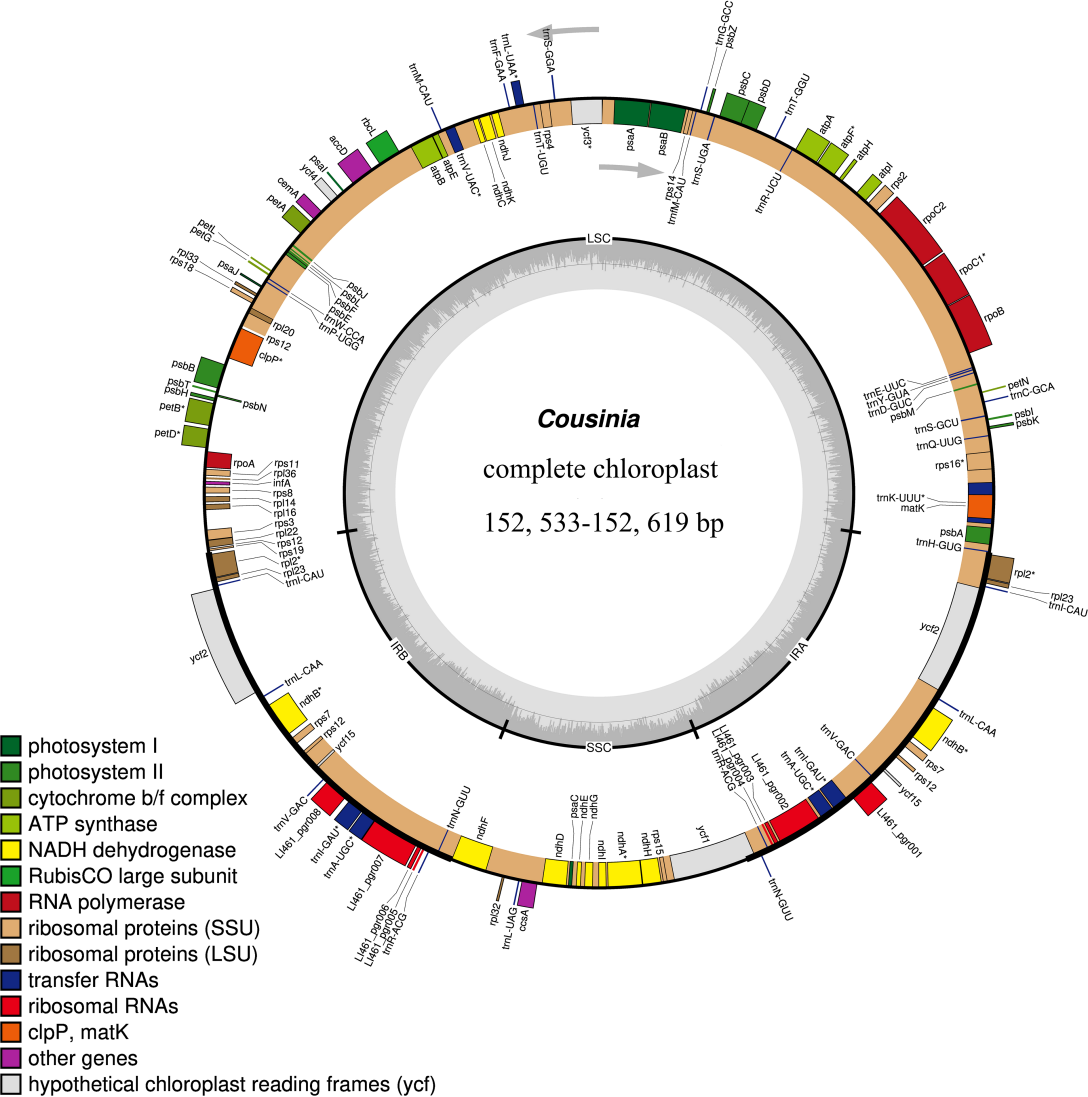
Gene map of the *Cousinia* chloroplast genome. Genes shown outside the outer circle are transcribed clockwise, and those insides are transcribed counterclockwise. Genes are color coded according to different functional groups. The darker gray in the inner circle indicates the GC content, and the lighter gray indicates the AT content. The inner circle also shows that the chloroplast genome contains two copies of inverted repeats (IRA and IRB), a large single-copy (LSC) region and a small single-copy (SSC)region.

We obtained the full cp genome sequences of the six *Cousinia* species ranged from 152,553 to 152,619 bp (**Figure 2**). The cp genome presented a typical quadripartite structure including one LSC region, one SSC region, and a pair of IR regions, respectively. The gene order within the genus was generally conserved, except for the SSC region, which exhibited a reverse orientation in the six *Cousinia* species, in contrast to the forward orientation observed in *C. thomsonii*. The GC content of the seven *Cousinia* species was nearly identical (**Table 1**).

**Table 1:** General characteristics of the plastomes of the seven *Cousinia* species included in this study.

Species	*Cousinia rhodantha*	*Cousinia rotundifolia*	*Cousinia pseudodshizakensis*	*Cousinia orthacantha*	*Cousinia subcandicans*	*Cousinia proxima*	*Cousinia thomsonii*
GenBank No.	PQ152229	PQ240609	PQ240610	PQ240608	PQ389802	PQ240605	PP525141^*^
Total length (bp)	152, 602	152, 559	152, 553	152, 554	152, 579	152, 619	152, 245
Length of LSC (bp)	83, 640	83, 608	83, 605	83, 689	83, 663	83, 725	83, 606
Length of SSC (bp)	18, 604	18, 593	18, 590	18, 530	18, 584	18, 564	18, 293
Length of IR (bp)	25, 179	25, 179	25, 179	25, 168	25, 175	25, 165	25, 173
Number of genes	131	131	131	131	131	131	132
Number of protein coding genes	87 (7)	87 (7)	87 (7)	87 (7)	87 (7)	87 (7)	88 (8)
Number of tRNA genes	36 (7)	36 (7)	36 (7)	36 (7)	36 (7)	36 (7)	36 (7)
Number of rRNA genes	8 (4)	8 (4)	8 (4)	8 (4)	8 (4)	8 (4)	8 (4)
Total GC content (%)	37.68	37.67	37.68	37.71	37.73	37.71	37.75
LSC GC content (%)	35.82	35.81	35.82	35.84	35.88	35.85	35.87
IR GC content (%)	43.10	43.09	43.10	43.11	43.11	43.12	43.12
SSC GC content (%)	31.36	31.35	31.38	31.49	31.46	31.47	31.55

^*^ Sequence downloaded from Gen Bank. Numbers in brackets indicate genes duplicated in the IR regions.

In six species 131 genes were annotated, of which 87 were protein-coding genes (PCGs), 36 were transfer RNA (tRNA) genes, and 8 were ribosomal RNA (rRNA) genes (**Table 2**). Notably, 18 genes within the cp genome are duplicated. This set of duplicated genes includes seven tRNAs (*trnA-UGC, trnI-CAU, trnI-GAU, trnL-CAA, trnN-GUU, trnR-ACG, trnV-GAC*), four rRNAs (*rrn16, rrn23, rrn4.5, rrn5*), and seven PCGs (*ndhB, rpl2, rpl23, rps12, ycf2, ycf15, rps7*). Sixteen genes possess introns, of which 11 are protein-coding genes (namely *atpF, rpoC1, rpl2, ndhB, ndhA, petB, petD, rps16, rps12, ycf3*, and *clpP*) and five are tRNA genes (specifically *tRNA-UGC, trnI-GAU, trnK-UUU, trnL-UAA*, and *trnV-UAC*). Two genes, *ycf3* and *clpP* have two introns, while the remaining 14 genes have only one.

**Table 2:** Gene composition of chloroplast genome of six *Cousinia* species.

Gene category	Gene group	Gene name
Self-replication	Lage subunit of ribosomal proteins	rpl2^*and+^, rpl14, rpl16, rpl20, rpl22, rpl23^*^, rpl32, rpl33, rpl36
	Small subunit of ribosomal proteins	rps2, rps3, rps4, rps7^*^ rps8, rps11, rps12^*and+^, rps14, rps15, rps16^+^, rps18, rps19
	DNA-dependent RNA polymerase	*rpoA, rpoB, rpoC1^+^, rpoC2*
	Ribosomal genes	rRNA 4.5S^*^, rRNA 5S^*^, rRNA 16S^*^ rRNA 23S^*^
	Transfer RNA genes	trnA-UGC^*and+^, trnC-GCA, trnD-GUC, trnE-UUC, trnF-GAA, trnfM-CAU, trnG-GCC, trnH-GUG, trnI-CAU^*^, trnI-GAU^*and+^, trnK-UUU^+^, trnL-CAA^*^, trnL-UAA^+^, trnL-UAG, trnM-CAU, trnN-GUU^*^, trnP-UGG, trnQ-UUG, trnR-ACG^*^, trnR-UCU, trnS-GCU, trnS-GGA, trnS-UGA, trnT-GGU, trnT-UGU, trnV-GAC^*^, trnV-UAC^+^, trnW-CCA, trnY-GUA
Gene for photosynthesis	Subunits of NADH dehydrogenase	ndhA^+^, ndhB^*and+^, ndhC, ndhD, ndhE, ndhF, ndhG, ndhH, ndhI, ndhJ, ndhK
	Photosystem I	*psaA, psaB, psaC, psaI, psaJ*
	Photosystem II	*psbA, psbB, psbC, psbD, psbE, psbF, psbH, psbI, psbJ, psbK, psbL, psbM, psbN, psbT, psbZ*
	Subunits of ATP synthase	*atpA, atpB, atpE, atpF^+^, atpH, atpI*
	Subunits of Cytochrome	*petA, petB^+^, petD^+^,petG, petL, petN*
	Large subunit of Rubisco	*rbcL*
Other genes	maturase	*matK*
	Protease	*clpP^++^*
	Envelope membrane protein	*cemA*
	Translation initiation factor	*infA*
	C-type cytochrome synthesis	*ccsA*
	Subunit of Acetyl CoA Carboxylase	*accD*
Genes of unknown function	Conserved hypothetical chloroplast	ycf1, ycf2^*^, ycf3^++^, ycf4, ycf15^*^,

*^+^* The gene containing a single intron.

*^++^* Gene containing two introns.

^*^ Two gene copies in IRs.

### Long repeat sequences and SSR analyses

**Figure 3:**
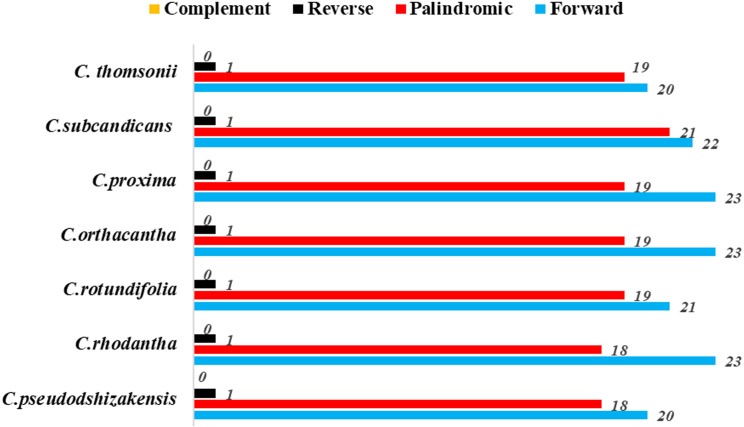
Number of long repetitive sequences in seven *Cousinia* species.

Among the seven plant species within the *Cousinia* genus, the number of long repeats ranged from 39 to 44 (**Figure 3**). The long repetitive sequences were primarily forward and palindromic repeats, with only a single reverse repeat identified. Of these repeats, the shortest is only 30 bp in length, while the longest is as large as 57 bp.

**Figure 4:**
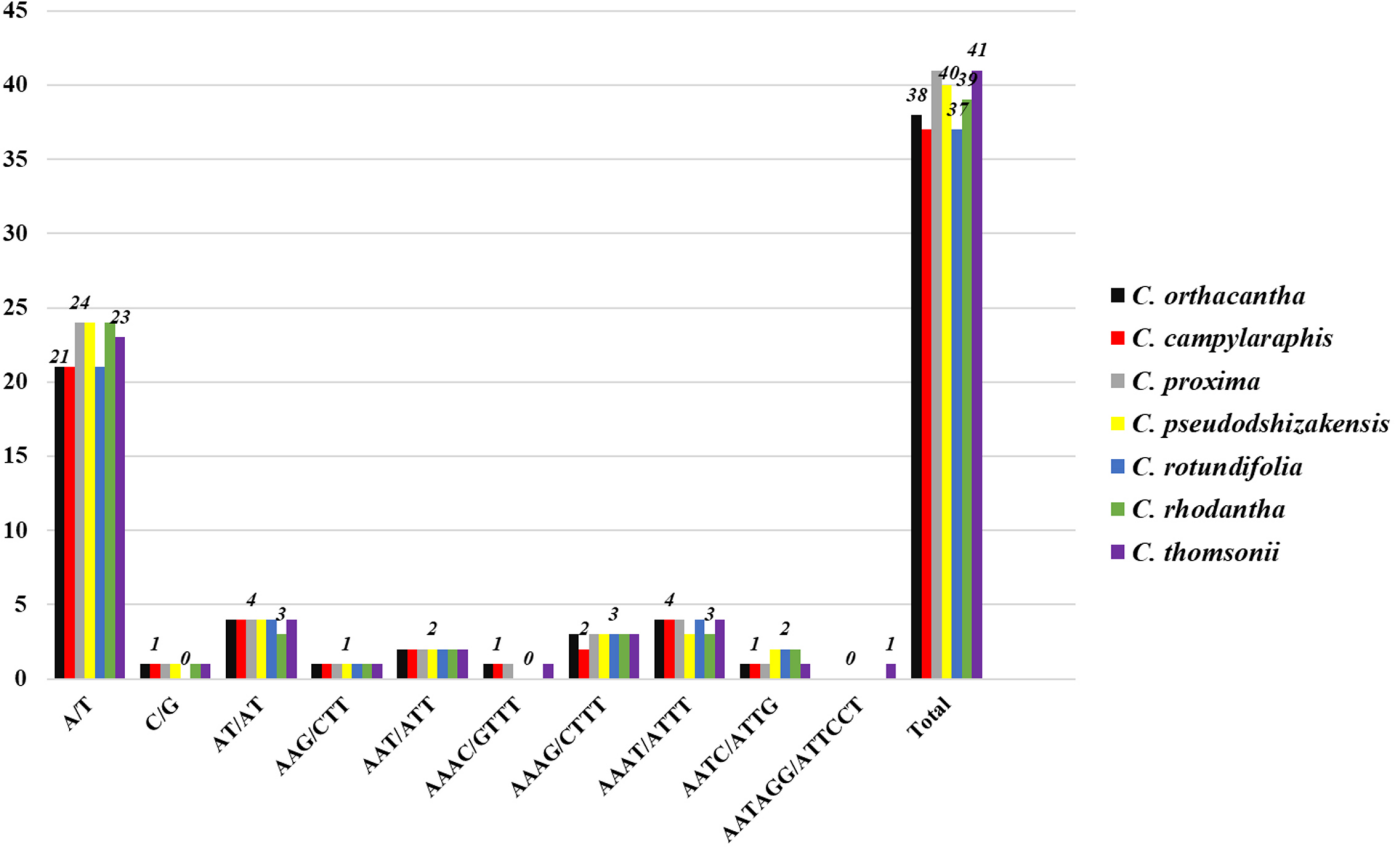
Number of SSR types detected in seven chloroplast genomes.

Five types of simple sequence repeats (SSRs) were identified: mononucleotide, dinucleotide, trinucleotide, tetranucleotide, and hexanucleotide. Among these, the A/T repeat was the most commonly observed across all species (**Figure 4**). The C/G repeat type was absent in *C. rotundifolia*, but it appeared once in each of the other species. A repeat type, AAAC/GTTT, was detected in *C. orthacantha*, *C. subcandicans*, and *C. proxima*, while the repeat type AATAGG/ATTCCT was found only in *C. thomsonii*. The majority of the identified SSRs were located in the LSC region of the genome.

### IR expansion and contraction

**Figure 5:**
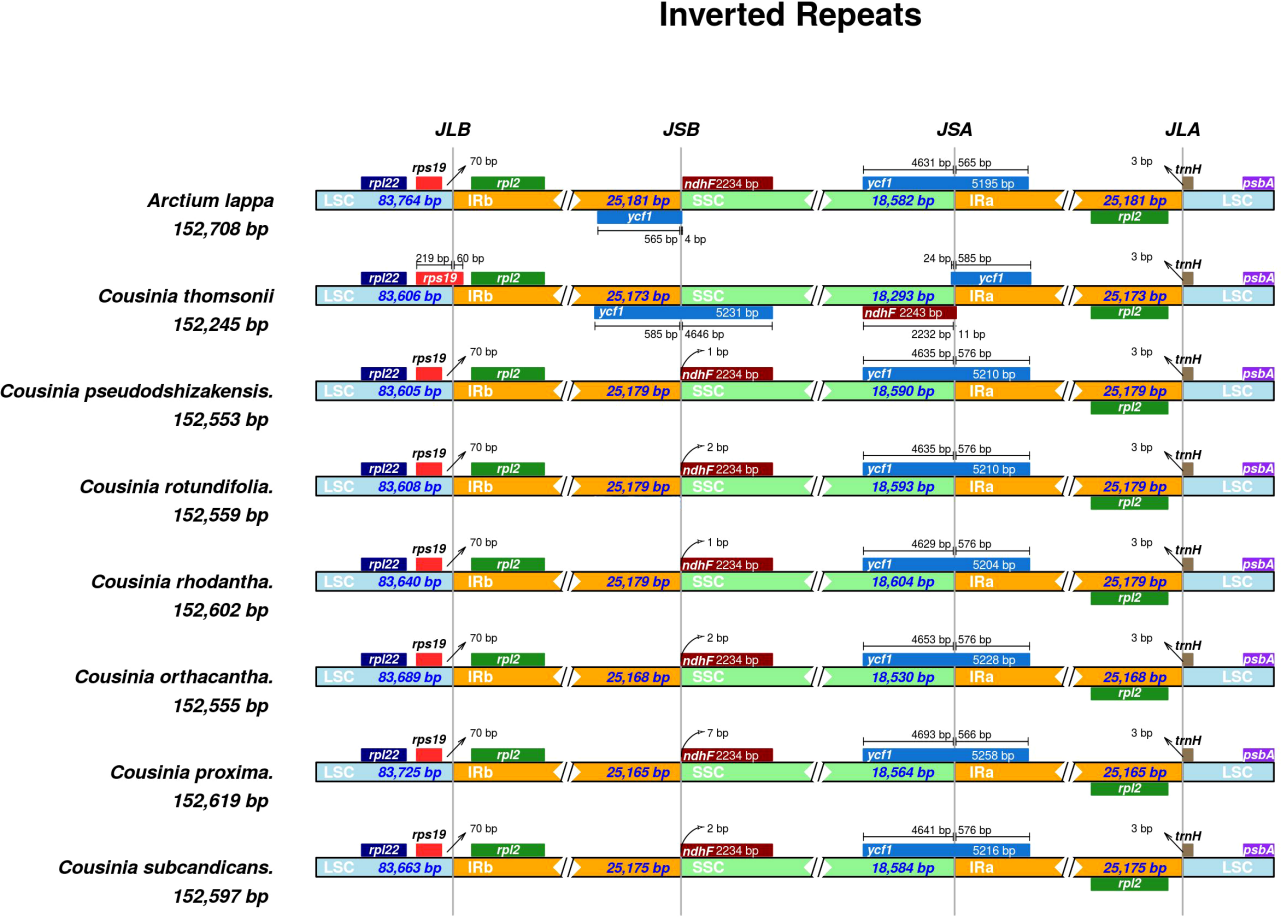
Comparisons of the borders of large single copy (LSC), small single copy (SSC), and inverted repeats IR regions (IR) among seven *Cousinia* species and *Arctium lappa*.

A comparison of Inverted Repeat (IR) region boundaries of *Cousinia* species with *Arctium lappa* revealed that they share a similar genomic arrangement. However, some differences were detected (**Figure 5**). In all *Cousinia* species, the *rps19* gene was found to be situated at the JLB (Large Single Copy and Inverted Repeat junction b), where it extended 60 base pairs into the IRb (Inverted Repeat b region). In contrast, in the case of *Arctium lappa*, the *rps19* gene was located in the Large Single Copy (LSC) region, 70 base pairs from the JLB junction. In *Arctium lappa*, the ycf1 gene had a duplicate copy, which was positioned at the JSB (Small Single Copy and Inverted Repeat b junction) and JSA (Small Single Copy and Inverted Repeat a junction). However, the *ycf1* gene was located only in JSA in all species of the *Cousinia* genus.

### Nucleotide diversity

**Figure 6:**
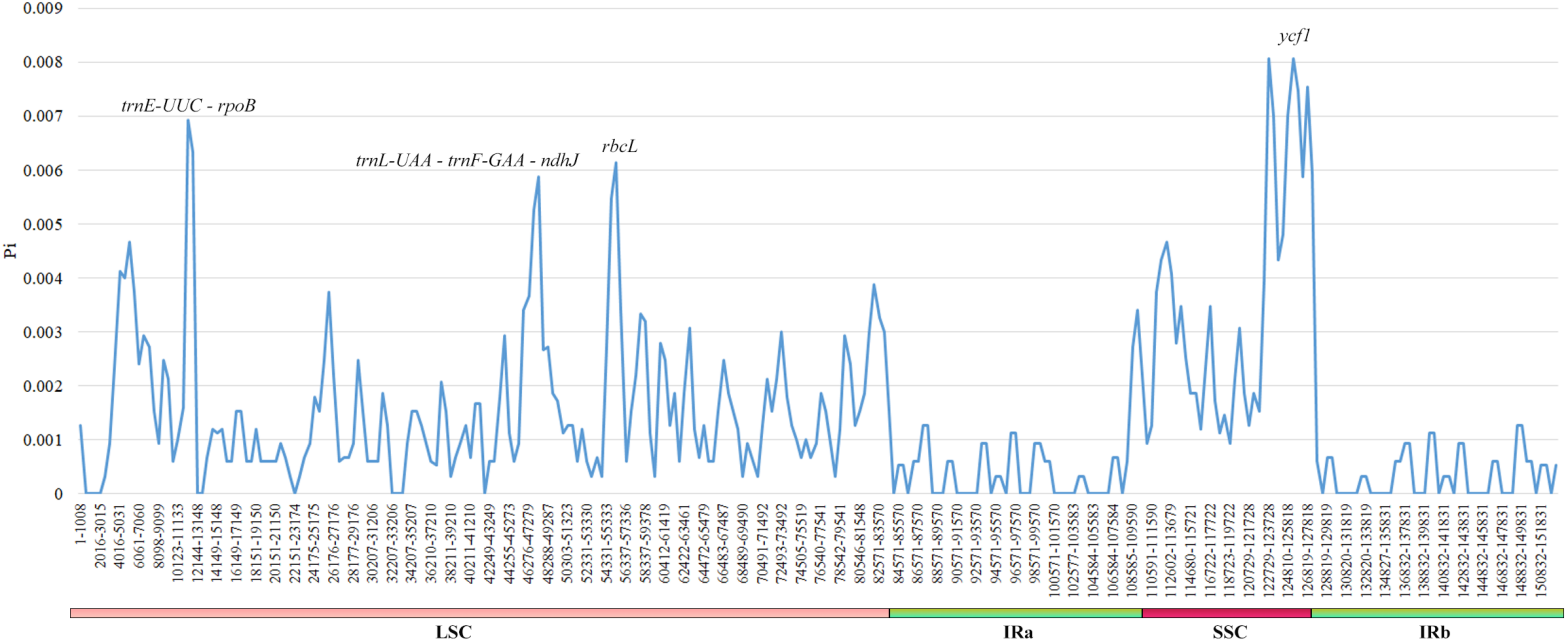
Comparison of the nucleotide variability (Pi) values between six *Cousinia* species.

The examination of nucleotide diversity in six *Cousinia* species showed that their cp genomes are highly conserved. From 152,892 nucleotide sites only 412 were polymorphic of which 259 were parsimony informative. Comparison of the cp genomes of two *C. orthacantha* specimens collected from populations inhabiting different ecological habitats revealed a single nucleotide difference (in the intron region of the *rps16* gene). The Pi values for six plastomes ranged from 0 to 0.00807 (**Figure 6**). Four regions *trnE-UUC – rpoB*, *trnL-UAA* – *trnF-GAA* – *ndhJ*, *rbcL* and *ycf1* exhibited Pi values greater than 0.005. The first three regions are situated in the LSC region of the plastome, while *ycf1*, located in the SSC region and approximately 6000 bp in length, had a Pi value of 0.00807. In contrast, all regions within the IR region had Pi values below 0.002.

### Phylogenetic analysis

**Figure 7:**
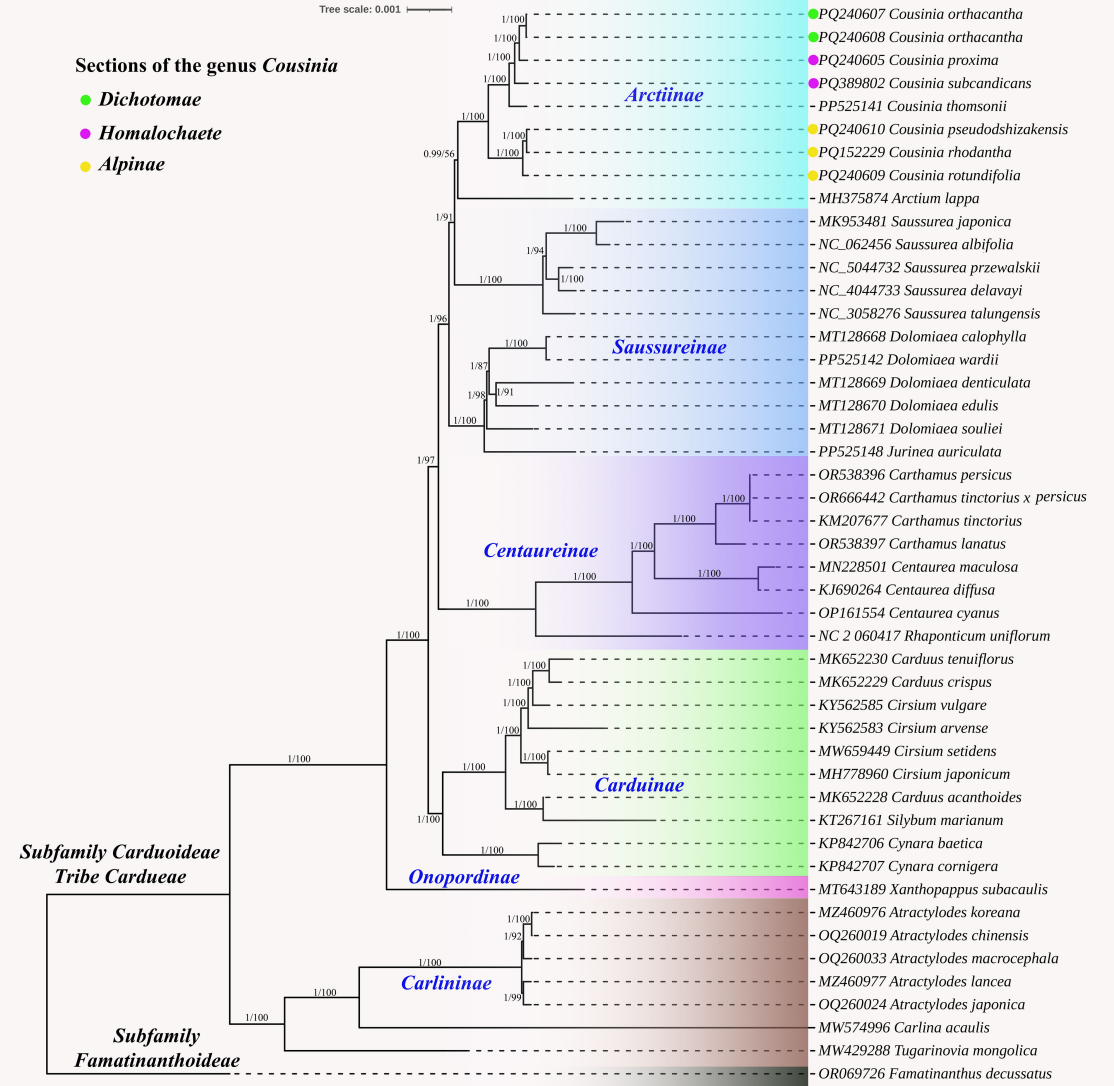
Phylogenetic tree constructed using the Bayesian inference (BI) and maximum likelihood (ML) methods based on the whole chloroplast genomes from 46 different species. The numbers near the branches represent the Bl posterior probabilities and MI bootstrap values.

The complete plastome-based trees obtained using the Bayesian Inference (BI) and Maximum Likelihood (ML) methods were congruent. Our tribal level tree (**Figure 7**) included six subtribes (Arctiinae, Saussureinae, Centaureinae, Carduinae, Onopordinae, and Carlininae) of Cardueae tribe. Except for Saussureinae, all subtribes formed monophyletic clusters. The Carlininae subtribe occupied a basal position. In the Arctiinae subtribe, the *Cousinia* species formed a monophyletic group with *Arctium lappa* from the *Arctium* genus (0.99/56).

The *Cousinia* species were divided into two distinct subclades. The first clade included *C. pseudodshizakensis, C. rhodantha, and C. rotundifolia*, which are part of the *Alpinae* section. The second clade consisted of *C. orthacantha, C. proxima, C. subcandicans*, and *C. thomsonii*. *C. orthacantha* belongs to the *Dichotomae* section, while *C. proxima* and *C. subcandicans* are part of the *Homalochaete* section.

**Figure 8:**
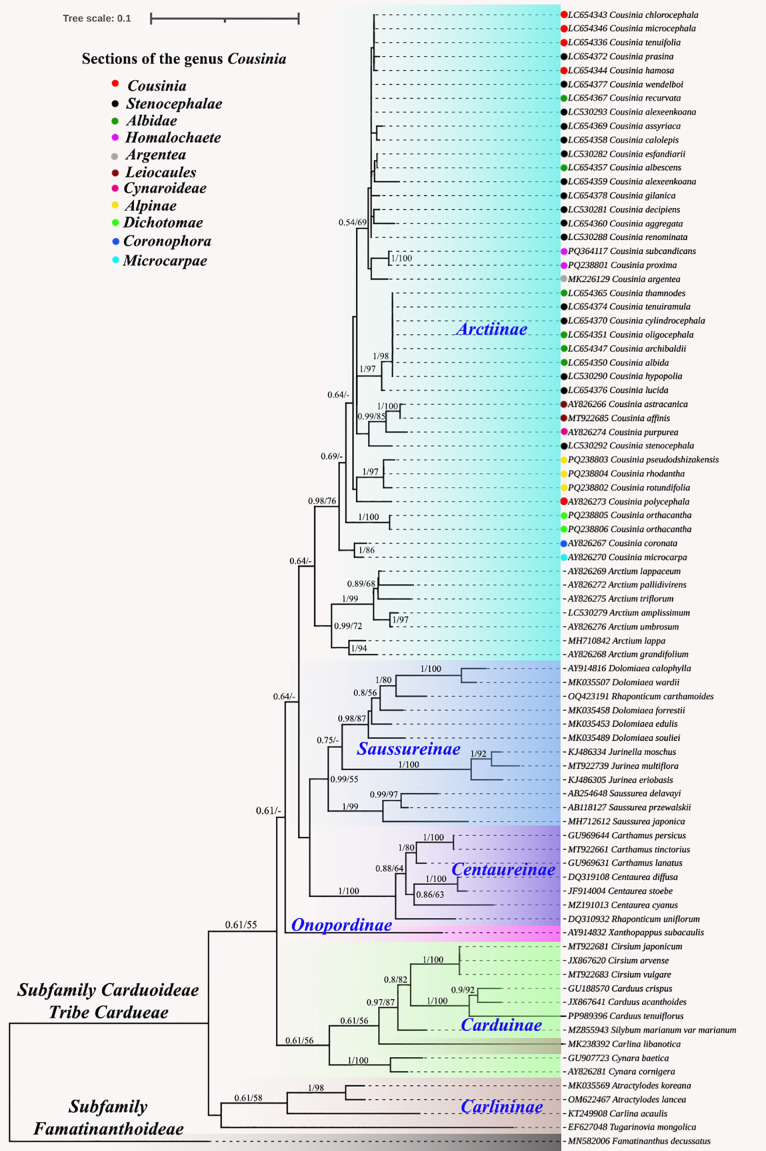
Phylogenetic tree constructed using the Bayesian inference (BI) and maximum likelihood (ML) methods based on the ITS from 80 different species. The numbers near the branches represent the Bl posterior probabilities and MI bootstrap values (below 0.52/52 not displayed).

Similarly, the nrDNA ITS based phylogeny recovered using BI and ML methods demonstrated congruence. However, while all subtribes except Carlininae formed monophyletic groups, *Carlina libanotica* Boiss., a member of the Carlininae subtribe, was unexpectedly positioned within the Carduinae subtribe (**Figure 8**). Within the Arctiinae subtribe, *Cousinia* species formed a well-supported monophyletic clade (0.98/76), clustering with *Arctium* species. The *Cousinia* clade was further subdivided into multiple subclades, however, some *Cousinia* species were not well resolved and did not correspond to their traditionally assigned taxonomic sections. Specifically, species from the *Stenocephalae*, *Albidae*, and *Cousinia* sections exhibited ambiguous phylogenetic placements, forming paraphyletic or polyphyletic groupings.

### Morphology of the anther appendages

**Figure 9:**
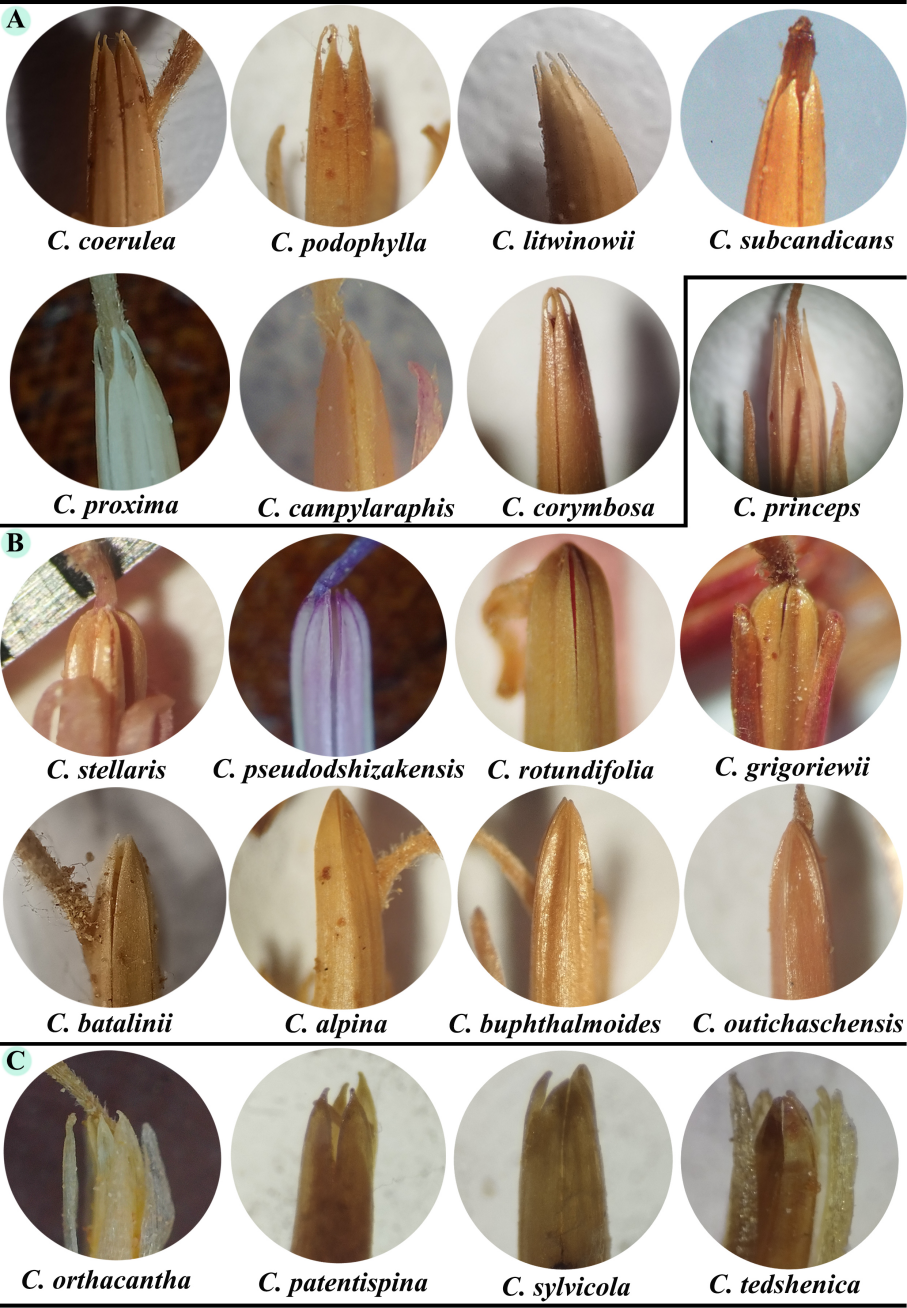
The form of the anther appendages in species from the **(A)** *Homalochaete*, **(B)** *Alpinae*, and **(C)** *Dichotomae* sections.

The shape of the anther appendages of 7 species from *Homalochaete*, 9 species from *Alpinae*, and 4 species from section *Dichotomae* were examined under a microscope. The findings indicated that in the *Homalochaete* section, the appendages all investigated species are characterized by a narrow, elongated, acute, and toothed shape. The *Alpinae* section features appendages that are short, acute, or obtuse, and have an arched outline. Except for *C. princeps*, the anther appendages are generally narrowly long and acute. In the *Dichotomae* section, the appendages are short, acute, and very short with a toothed appendage (**Figure 9**).

## Discussion

In this research, we performed sequencing of six species of *Cousinia* and carried out a comparative analysis of their cp genomes. This is the first report of complete cp. genomes from the genus *Cousinia*. They were found to be similar to those of other genera in Asteraceae with typical double-stranded circular tetramer structures and sizes ranging from 152,555 bp to 155,619 bp (Xing et al., 2019; Wu et al., 2019; Yun and Kim, 2022; He et al., 2023). The cp. genomes of six *Cousinia* species encoded 131 genes like most other species of Asteraceae, pseudogenes *ycf1* and *rps19* were also detected (Liu et al., 2024). A total of 16 intron-containing genes were identified in six plants of the genus *Cousinia*. These included 14 genes with one intron, and two genes with two introns. These intron-containing genes can play important roles in regulating gene expression (Wu et al., 2023). The total GC contents of the complete cp. genomes among the seven species of the *Cousinia* varied 37.67 to 37.75 (**Table 1**). Among the LSC, SSC, and IR regions, the IR regions had the highest GC contents (43.09 to 43.12%), followed by the LSC (35.81 to 35.88) and SSC regions (31.35 to 31.55%). The IR regions had the highest GC contents among the four regions, which is likely due to the presence of two copies of rRNAs (*rrna4.5, rrna5, rrna23*, and *rrna16*) within this region (Shahzadi et al., 2020; Liu et al., 2024).

In this study, the repetitive sequences observed in the cp genomes of the seven *Cousinia* plant species displayed no significant heterogeneity in terms of their number, type, or length. Among the repetitive sequence types, reverse repeats were limited to only one in all species, while palindromic (P) repeats ranged from 18 to 21, and forward (F) repeats ranged from 20 to 23. Notably, complementary (C) repeats were absent in all seven *Cousinia* species. These long repetitive sequences play crucial roles in gene recombination and sequence structure variation (He et al., 2023). Furthermore, they may serve as valuable markers for differentiating *Cousinia* sp*ecies*, offering potential applications in species identification and phylogenetic studies (Gao et al., 2018).

SSRs, also called microsatellites, are widely utilized as valuable molecular markers in species identification and phylogenetic studies due to their high substitution rates (Upadhyay et al., 2010). In this study, a total of 273 SSRs were identified in the complete cp. genomes of seven *Cousinia* species, 158 were found to be mononucleotide repeats, constituting the majority (57.8%) of all SSRs (**Figure 4**). Previous studies have suggested that this may be related to natural selection and genetic mutation (He et al., 2023; Zhang et al., 2023).

Vascular plant cp genomes usually exhibit the contraction or expansion of boundaries within different genera or even within the same genus, which is the main factor leading to variations in the length and number of genes in various species (Gu et al., 2024). The LSC/IRb boundary was generally located between the *rps19* and *rpl2* genes in the plastid genomes of six *Cousinia* species and *Arctium lappa* (NC042724). However, in *Cousinia thomsonii* (PP525141), the boundary was located within the *rps19* gene. Upon further analysis of *rps19* using Geneious v9.0.2, an illegal start codon was detected, suggesting potential annotation or sequencing issues that require further investigation. Moreover, the orientation of the SSC region in *C. thomsonii* was found to be forward, in contrast to the reverse orientation observed in other *Cousinia* species and *A. lappa*. This unique structural variation highlights the genomic divergence within the group. In *C. thomsonii* and *A. lappa*, the expansion of the IR region resulted in partially duplicated *ycf1* genes being located in the IRb region, thereby generating a pseudogene *ycf1* at the IRb/SSC boundary. While most land plants typically have pseudogenes in their cp genomes at the SSC/IRa boundary (Cai et al., 2023), the six *Cousinia* species analyzed did not exhibit annotated pseudogenes at this boundary. This absence is likely due to nucleotide insertions or deletions (indels) that disrupted the formation of a stop codon, preventing the annotation of pseudogenes in these regions.

Cp genomes provide essential resources for phylogenetic and taxonomic studies due to their conserved structure and variability in specific regions. In this study, we identified regions with nucleotide diversity (Pi) values exceeding 0.005, including *trnE-UUC – rpoB*, *trnL-UAA – trnF*-*GAA – ndhJ*, *rbcL*, and *ycf1*. These highly variable regions represent potential molecular markers for resolving phylogenetic relationships within the genus *Cousinia*. Previous studies have also demonstrated the effectiveness of cp markers such as *trnL-trnF*, *trnL-trnT-rps4*, and *rpl32-trnL* in differentiating *Cousinia* species (Susanna et al., 2006; López-Vinyallonga et al., 2009, 2011). The results highlight the utility of cp genome variability for species-level discrimination and evolutionary studies in *Cousinia*.

The phylogenetic analysis of the complete plastomes demonstrated congruent topologies, underscoring the reliability of plastome data for resolving phylogenetic relationships within the Cardueae tribe. One of the notable findings is the monophyletic clustering of most subtribes, with the exception of Saussureinae, indicating that this subtribe might be polyphyletic. This result aligns with previous studies suggesting complexities in Saussureinae’s taxonomy and phylogenetic placement (Herrando-Moraira et al., 2020). The basal position of the Carlininae subtribe is consistent with earlier phylogenetic reconstructions, supporting its potential as an ancestral lineage within Cardueae (Herrando-Moraira et al., 2019). Within the Arctiinae subtribe, the monophyletic grouping of *Cousinia* species with *Arctium lappa* supports previous findings by López-Vinyallonga et al. (2009, 2011), who identified a close phylogenetic relationship between these genera.

In this study, we investigated the molecular and morphological distinctions within *Cousinia*, particularly focusing on anther appendage shape as a taxonomic trait, alongside molecular phylogenetic data. Our results revealed two major molecular clades among the *Cousinia* species, which show some alignment with traditional sectional classifications, though with notable exceptions and inconsistencies. The first molecular clade, which includes *C. pseudodshizakensis*, *C. rhodantha*, and *C. rotundifolia*, corresponds to the *Alpinae* section. This molecular grouping is consistent with morphological observations, where species in the *Alpinae* section exhibit short, acute or obtuse anther appendages with an arched outline. However, the morphological data also suggest that *C. princeps* from the *Alpinae* section deviates in its anther appendage shape, which aligns more closely with species in the *Tianschanicae* and *Subappendiculatae* sections. This discrepancy indicates that while anther appendage morphology provides useful diagnostic features, it may not be entirely sufficient to classify species at a sectional level, especially when taxonomic boundaries overlap. The second molecular clade consists of species from the *Dichotomae* and *Homalochaete* sections, including *C. orthacantha*, *C. proxima*, *C. subcandicans*. Morphologically, these species share similar anther appendage traits narrow, elongated, acute, and toothed appendages in the *Homalochaete* section and short, acute, toothed appendages in the *Dichotomae* section. The congruence between molecular data and the anther appendage morphology for these species supports the close evolutionary relationship between the *Dichotomae* and *Homalochaete* sections. This finding is consistent with previous work by Rechinger (1972) and Sennikov (2010), who highlighted the taxonomic significance of anther appendage morphology in *Cousinia*. These traits serve as valuable markers in identifying evolutionary relationships within sections. To resolve the current uncertainties in the intersectional classification of *Cousinia*, particularly concerning the *Alpinae, Tianschanicae*, and *Subappendiculatae* sections, further molecular and morphological analyses are essential.

The comparative analysis of nrDNA ITS sequences, cp data, and morphological traits provided critical insights into the phylogenetic relationships within *Cousinia*. While plastome phylogenies demonstrated stable and congruent topologies, supporting well-defined monophyletic clades, ITS based phylogenies were largely consistent with plastome data, with only *Carlina libanotica* unexpectedly positioned within the Carduinae subtribe rather than Carlininae. This anomaly highlights the limitations of ITS as a sole marker for species delimitation. In particular, taxa belonging to the groups *Stenocephalae, Albidae*, and *Cousinia* had unclear phylogenetic positions, establishing paraphyletic or polyphyletic groupings. These results are consistent with earlier studies by Kalouti et al. (2022), which also reported evolutionary complexity in these areas.

Morphological data, particularly anther appendage shape, showed partial alignment with molecular clades, reinforcing its taxonomic relevance. For instance, the *Alpinae* section displayed relatively strong molecular-morphological congruence. Similarly, species from the *Dichotomae* and *Homalochaete* sections exhibited consistent molecular clustering, with anther appendage morphology further supporting their evolutionary affinity. These findings highlight the necessity of an integrative taxonomic approach, where plastome data offer robust phylogenetic resolution, ITS markers capture finer-scale divergence but require cautious interpretation, and morphological traits provide critical but sometimes homoplastic diagnostic features. A combined strategy incorporating plastome, nuclear, and morphological data is essential for refining the taxonomy and phylogenetic framework of *Cousinia.*

## Conclusion

This study compares the entire cp genome sequences within *Cousinia* genus for the first time. There were 131 genes found in the cp genomes of six different *Cousinia* species, which varied in size from 152,553 to 152,619 bp. Higher nucleotide polymorphism levels were found in *trnE-UUC – rpoB, trnL-UAA – trnF-GAA – ndhJ, rbcL*, and *ycf1* genomic regions, indicating possible markers for species differentiation. Within *Cousinia*, two different clades were identified by phylogenetic analysis using whole cp genomes. In addition, anther appendage morphology investigation revealed *C. princeps* from *Alpinae* section would need to be reclassified. Additionally, the phylogenetic analysis of the *Cardueae* tribe and its subtribes, along with the subtribal relationships within the genus, suggests that cp genome data holds significant potential for revising evolutionary and taxonomic frameworks within both *Cousinia* and broader Asteraceae studies. These results are preliminary because of the small number of species that were examined. Enhancing the intersectional classification within *Cousinia* requires additional study using a larger sample size.

## Data Availability

The datasets presented in this study can be found in online repositories. The names of the repository/repositories and accession number(s) can be found in the article/**Supplementary Material**.

## Supplementary material

Supplementary Table 1 The information about sampled specimens.

Supplementary Table 2 Gen Bank accession numbers of sampled specimens.

Supplementary Table 3 Information of the cp and nrDNA ITS sequences used in this study.
