# Supplementary material for: Comparative analysis of complete chloroplast genomes of Cousinia (Asteraceae) species
Source: Front Plant Sci. 2025 Apr 29;16:1522950. doi: 10.3389/fpls.2025.1522950 (PMC12069278; doi:10.3389/fpls.2025.1522950)
Supplement: Supplementary Table 1 — The information about sampled specimens. [file Table1.doc]

**Supplementary table 1.** The information about sampled specimens

| **Species** | **Collected place** | **Collected date** | | **Identifed** | **Voucher** | |  |
| --- | --- | --- | --- | --- | --- | --- | --- |
| *C.proxima* | Uzbekistan, Sangardak waterfall | 2023 | K.Bobur | | | C2 | |
| *C. subcandicans* | Uzbekistan, Hisar state reserve | 2023 | K.Bobur | | | C3 | |
| *C.orthacantha* | Uzbekistan, Sherabad valley | 2023 | K.Bobur | | | C4 | |
| *C. orthacantha* | Uzbekistan, Sherabad valley | 2023 | K.Bobur | | | C5 | |
| *C. rhodantha* | Uzbekistan, S-W Hissar, Baisun | 2023 | A.Gaziyev | | | C6 | |
| *C. rotundifolia* | Uzbekistan, S-W Hissar, Baisun | 2023 | A.Gaziyev | | | C7 | |
| *C. pseudodshizakensis* | Uzbekistan, Molguzar mountain | 2023 | N.Beshko | | | C8 | |
