# Supplementary material for: Comparative analysis of complete chloroplast genomes of Cousinia (Asteraceae) species
Source: Front Plant Sci. 2025 Apr 29;16:1522950. doi: 10.3389/fpls.2025.1522950 (PMC12069278; doi:10.3389/fpls.2025.1522950)
Supplement: Supplementary Table 2 — Gen Bank accession numbers of sampled specimens. [file Table2.docx]

**Supplementary table 2.** Gen Bank accession numbers of sampled specimens

| **Species** | **Generated paired-end reads** | **SRA** | **Biosample** | **cp** | **ITS** |
| --- | --- | --- | --- | --- | --- |
| *C. proxima* | 8,983,956 | SRR29430600 | SAMN41862839 | PQ240605 | PQ238801 |
| *C. subcandicans* | 20,105,500 | - | - | PQ389802 | PQ364117 |
| *C. orthacantha* | 7,981,643 | SRR29456473 | SAMN41893244 | PQ240607 | PQ238806 |
| *C. orthacantha* | 8,100,072 | SRR29446580 | SAMN41885406 | PQ240608 | PQ238805 |
| *C. rhodantha* | 10,322,054 | SRR29456472 | SAMN41885987 | PQ152229 | PQ238804 |
| *C. rotundifolia* | 9,302,278 | SRR29456471 | SAMN41885988 | PQ240609 | PQ238802 |
| *C. pseudodshizakensis* | 7,890,409 | SRR29456470 | SAMN41885989 | PQ240610 | PQ238803 |
