# Supplementary material for: Comparative analysis of complete chloroplast genomes of Cousinia (Asteraceae) species
Source: Front Plant Sci. 2025 Apr 29;16:1522950. doi: 10.3389/fpls.2025.1522950 (PMC12069278; doi:10.3389/fpls.2025.1522950)
Supplement: Supplementary Table 3 — Information of the cp and nrDNA ITS sequences used in this study. [file Table3.docx]

**Supplementary table 3**. Information of the cp and nrDNA ITS sequences used in this study

| **Subtribe** | **Species** | | **Accesion No.cp** | **Accesion No. nrDNA ITS** |
| --- | --- | --- | --- | --- |
| Arctiinae N.Garcia & Susanna, 2019 | *Cousinia orthacantha* Tscherneva | | PQ240607 | PQ238805 |
|  | *Cousinia orthacantha* Tscherneva | | PQ240608 | PQ238806 |
|  | *Cousinia proxima* Juz. | | PQ240605 | PQ238801 |
|  | *Cousinia subcandicans* Tschern. | | PQ389802 | PQ364117 |
|  | *Cousinia thomsonii* C.B.Clarke | | PP525141 | // |
|  | *Cousinia rhodantha* Kult. | | PQ152229 | PQ238804 |
|  | *Cousinia rotundifolia* C.Winkl. | | PQ240609 | PQ238802 |
|  | *Cousinia pseudodshizakensis* Tschern. & Vved. | | PQ240610 | PQ238803 |
|  | *Cousinia thamnodes* Boiss. & Hausskn. | | // | LC654365 |
|  | *Cousinia tenuiramula* | | // | LC654374 |
|  | *Cousinia oligocephala* | | // | LC654351 |
|  | *Cousinia cylindrocephala* | | // | LC654370 |
|  | *Cousinia archibaldii* | | // | LC654347 |
|  | *Cousinia hypopolia* | | // | LC530290 |
|  | *Cousinia albida* | | // | LC654350 |
|  | *Cousinia lucida* | | // | LC654376 |
|  | *Cousinia gilanica* | | // | LC654378 |
|  | *Cousinia wendelboi* | | // | LC654377 |
|  | *Cousinia recurvata* | | // | LC654367 |
|  | *Cousinia tenuifolia* | | // | LC654336 |
|  | *Cousinia chlorocephala* | | // | LC654343 |
|  | *Cousinia microcephala* | | // | LC654346 |
|  | *Cousinia prasina* | | // | LC654372 |
|  | *Cousinia hamosa* | | // | LC654344 |
|  | *Cousinia alexeenkoana* | | // | LC530293 |
|  | *Cousinia calolepis* | | // | LC654358 |
|  | *Cousinia esfandiarii* | | // | LC530282 |
|  | *Cousinia albescens* | | // | LC654357 |
|  | *Cousinia assyriaca* | | // | LC654369 |
|  | *Cousinia glaucopsis* | | // | LC654373 |
|  | *Cousinia aggregata* | | // | LC654360 |
|  | *Cousinia alexeenkoana* | | // | LC654359 |
|  | *Cousinia decipiens* | | // | LC530281 |
|  | *Cousinia renominata* | | // | LC530288 |
|  | *Cousinia argentea* | | // | MK226129 |
|  | *Cousinia microcarpa* | | // | AY826270 |
|  | *Cousinia coronata* | | // | AY826267 |
|  | *Cousinia purpurea* | | // | AY826274 |
|  | *Cousinia astracanica* | | // | AY826266 |
|  | *Cousinia affinis* | | // | MT922685 |
|  | *Cousinia stenocephala* | | // | LC530292 |
|  | *Cousinia polycephala* | | // | AY826273 |
|  | *Arctium umbrosum* | | // | AY826276 |
|  | *Arctium triflorum* | | // | AY826275 |
|  | *Arctium lappaceum* | | // | AY826269 |
|  | *Arctium amplissimum* | | // | LC530279 |
|  | *Arctium pallidivirens* | | // | AY826272 |
|  | *Arctium lappa* | | MH378874 | MH710842 |
|  | *Arctium grandifolium* | | // | AY826268 |
|  | *Saussurea japonica* (Thunb.) DC. | | MK953481 | MH712612 |
|  | *Saussurea albifolia M.J.Nam & Im* | | NC062456 | // |
|  | *Saussurea delavayi* Franch. | | NC4044733 | AB254648 |
|  | *Saussurea przewalskii* Maxim. | | NC5044732 | AB118127 |
|  | *Saussurea talungensis* S.K.Ghimire & H.K.Rana is a synonym of *Saussurea roylei* (DC.) Sch.Bip. | | NC3058276 | // |
|  | *Dolomiaea calophylla* Y.Ling | | MT128668 | AY914816 |
|  | *Dolomiaea wardii* (Hand.-Mazz.) Y.Ling | | PP525142 | MK035507 |
|  | *Dolomiaea denticulata* (Y.Ling) C.Shih is a synonym of *Dolomiaea forrestii* (Diels) C.Shih | | MT128669 | MK035458 |
|  | *Dolomiaea edulis* (Franch.) C.Shih | | MT128670 | MK035453 |
|  | *Dolomiaea souliei* (Franch.) C.Shih | | MT128671 | MK035489 |
|  | *Jurinea auriculata* (DC.) N.Garcia, Herrando & Susanna | | PP525148 | // |
|  | *Jurinea multiflora* | | // | MT922739 |
|  | *Jurinella moschus* | | // | KJ486334 |
|  | *Jurinea eriobasis* | | // | KJ486305 |
| Centaureinae Dumort., 1827 | *Carthamus persicus* Desf. ex Willd. | | OR538396 | GU969644 |
|  | *Carthamus tinctorius x Carthamus persicus* | | OR666442 |  |
|  | *Carthamus tinctorius* L. | | KM207677 | MT922661 |
|  | *Carthamus lanatus* L. synonym of *Phonus lanatus* (L.) Hill | | OR538397 | GU969631 |
|  | *Centaurea maculosa* Lam. synonym of *Centaurea stoebe* subsp. *stoebe* | | MN228501 | // |
|  | *Centaurea stoebe* | | // | JF914004 |
|  | *Centaurea diffusa* Lam. | | KJ690264 | DQ319108 |
|  | *Centaurea* *cyanus* L. | | OP161554 | MZ191013 |
|  | *Rhaponticum uniflorum* (L.) DC. | | NC2060417 | DQ310932 |
|  | Rhaponticum carthamoides | | // | OQ423191 |
| Carduinae Dumort., 1827 | *Carduus tenuiflorus*Curtis | | MK652230 | PP989396 |
|  | *Carduus crispus*L. | | MK652229 | GU188570 |
|  | *Carduus acanthoides L.* | | MK652228 | JX867641 |
|  | *Cirsium vulgare*(Savi) Ten. | | KY562585 | MT922683 |
|  | *Cirsium arvense*(L.) Scop. | | KY562583 | JX867620 |
|  | *Cirsium setidens*(Dunn) Nakai | | MW659449 | // |
|  | *Cirsium japonicum*DC. | | MH778960 | MT922681 |
|  | *Silybum marianum*(L.) Gaertn. | | KT267161 | // |
|  | *Silybum marianum* var. *marianum* | | // | MZ855943 |
|  | *Cynara baetica*(Spreng.) Pau | | KP842706 | GU907723 |
|  | *Cynara cornigera*Lindl. | | KP842707 | AY826281 |
| Onopordinae N.Garcia & Susanna, 2019 | *Xanthopappus subacaulis*C.Winkl. | | MT643189 | AY914832 |
| Carlininae Dumort., 1827 | *Atractylodes koreana*(Nakai) Kitam. | | MZ460976 | MK035569 |
|  | *Atractylodes chinensis*(Bunge) Koidz*.* synonym of *Atractylodes lancea*(Thunb.) DC*.* | | OQ260019 | // |
|  | *Atractylodes macrocephala*Koidz. | | OQ260033 |  |
|  | *Atractylodes lancea*(Thunb.) DC. | | MZ460977 | OM622467 |
|  | *Atractylodes japonica*Koidz. ex Kitam. synonym of *Atractylodes lancea* (Thunb.) DC. | | OQ260024 | // |
|  | *Carlina acaulis*L. | | MW429288 | KT249908 |
|  | *Carlina libanotica* | | // | MK238392 |
|  | *Tugarinovia mongolica*Iljin | | OR069726 | EF627048 |
| Outgroup | *Famatinanthus decussatus* | | OR069726 | MN582006 |
|  | |  |  |  |
